# Improving Uptake of Postnatal Checking of Blood Glucose in Women Who Had Gestational Diabetes Mellitus in Universal Healthcare Settings: A Systematic Review

**DOI:** 10.3390/jcm8010004

**Published:** 2018-12-20

**Authors:** Helen Sanderson, Emma Loveman, Jill Colquitt, Pamela Royle, Norman Waugh, Bee Kang Tan

**Affiliations:** 1 Warwick Medical School, University of Warwick, Coventry CV4 7AL, UK; helen.sanderson@alderhey.nhs.uk (H.S.); p.l.royle@warwick.ac.uk (P.R.); norman.waugh@warwick.ac.uk (N.W.); 2 Effective Evidence LLP, Waterlooville PO8 9SE, UK; emma.loveman@effectiveevidence.org (E.L.); jill.colquitt@effectiveevidence.org (J.C.); 3 Department of Cardiovascular Sciences and Leicester Diabetes Centre, University of Leicester, Leicester LE2 7LX, UK; 4 Department of Obstetrics and Gynaecology, University Hospitals of Leicester NHS Trust, Leicester LE1 5WW, UK; 5 Department of Obstetrics and Gynaecology, University Hospitals Birmingham NHS Foundation Trust, Birmingham B9 5SS, UK

**Keywords:** gestational diabetes mellitus, barriers, interventions, postnatal screening, systematic review

## Abstract

The aim of this systematic review is to look at the barriers to uptake and interventions to improve uptake of postnatal screening in women who have had gestational diabetes mellitus (GDM). Increasing postnatal screening rates could lead to timely interventions that could reduce the incidence of type 2 diabetes mellitus (T2DM), the associated long-term health complications, and the financial burden of T2DM. A systematic review of the literature was undertaken. PubMed, Embase, Medline, CINAHL and the Cochrane library databases were searched using well-defined search terms. Predefined inclusion and exclusion criteria were used to identify relevant manuscripts. Data extractions and quality assessments were performed by one reviewer and checked by a second reviewer. Eleven primary studies of various research design and three systematic reviews were included. We identified seven themes within these studies and these were described in two categories, barriers and interventions. There appeared to be no single intervention that would overcome all the identified barriers, however, reminders to women and healthcare professionals appear to be most effective. Uptake rates of testing for T2DM are low in women with GDM. Interventions developed with consideration of the identified barriers to uptake could promote greater numbers of women attending for follow-up.

## 1. Introduction

Gestational diabetes mellitus (GDM) affects 1–14% of all pregnancies globally [1]. The rate is increasing in the United Kingdom (UK) and worldwide [2,3,4], with estimated rates increasing from 4.6% to 9.2% in the United States of America (USA), and from 1% to 28% in other countries, though this is partly due to changing definitions [2,3]. In the UK and Ireland, the prevalence of GDM ranges from 1% to 24% depending upon the GDM criteria used [5].

GDM is not just a pregnancy complication but also predicts a high risk of developing type 2 diabetes mellitus (T2DM) subsequently. Up to 10% of women with presumed GDM are now being diagnosed with T2DM soon after delivery, implying that they had undiagnosed T2DM before pregnancy [6]. In long term studies, up to 70% of women with GDM developed T2DM by 10 years of follow-up [7]. Lifestyle changes and medications can reduce the incidence of T2DM in those with GDM [8,9]. However, for these to be effective, there is a need for:

- Thorough screening for GDM (prevention cannot start until the high-risk groups have been identified).
- Postnatal follow-up of all women with GDM (both short and long term) [9].

The 75g oral glucose tolerance test (OGTT) is the test recommended by the International Association of Diabetes and Pregnancy Study Groups and the American Diabetes Association [10,11]. The National Institute for Health and Care Excellence (NICE) 2008 guideline recommends that women with GDM should have postnatal follow-up with an OGTT screening test to determine if she has, or is at risk of, developing T2DM [12]. The recent NICE 2015 guideline has been amended and it is now recommended that women with GDM should have a fasting plasma glucose test 6 to 13 weeks after giving birth to exclude T2DM or if a fasting plasma glucose test has not been performed by 13 weeks, a later fasting plasma glucose test or a glycated haemoglobin test (if a fasting plasma glucose test is not possible) should be offered [4]. If the test is positive for T2DM or impaired glucose tolerance (IGT) then the woman can be treated, either with a glucose lowering diet and drugs if diabetic, or with an intervention to prevent diabetes if the test shows IGT. If the test is negative, then a yearly repeat of the test is recommended. Testing with fasting plasma glucose could be more convenient for the patient when compared to the OGTT, and screening rates for postnatal hyperglycaemia and T2DM could be enhanced [13]; however, fasting plasma glucose lacks sensitivity [14].

Keely [15] argues for screening, early identification, and interventions such as lifestyle changes, including exercise and nutritional advice through postnatal education. This can reduce the incidence and burden of T2DM. However, uptake of screening for T2DM in the postnatal period has been poor. In the UK, T2DM following GDM is on the increase [16,17].

T2DM can affect a woman’s health if not diagnosed and controlled, with an increased risk of macro and microvascular complications and potentially mortality [18]. Therefore, detection of continuing hyperglycaemia after delivery through screening in the postnatal period is highly desirable to detect those at high risk of later T2DM. A French study monitoring 49,080 women over 6 years from 2007 to 2013 found that only 18.5% of women had the recommended OGTT in 2007 by 3 months postnatal with no significant increase by 2013; rates increased slightly by 12 months with 46.8% having the test in 2007 and 54.1% in 2013; these numbers remain far below optimum [19].

Postnatal screening for T2DM in women with GDM has also been poor in the UK. McGovern et al. [16] found that only 18.5% of 788 women in the UK had a postnatal follow-up before 6 months. This improved slightly to 26.2% by 12 months postnatal. Similarly, Keely [15] states how repeated studies have shown uptake rates to be poor. A cohort study of 924,873 women in the USA showed an uptake rate of only 19% for postnatal screening [15].

Thus, the uptake of postnatal OGTT by women with GDM is poor. Why women do not attend this follow-up is not well understood and it is therefore hard to develop effective interventions to increase rates. The risk of developing T2DM and associated health complications is life-long, not just in the postnatal period. This systematic review seeks to discover both what stops women and what will encourage women with GDM within the National Health Service (NHS), UK, and similar healthcare systems, to attend postnatal screening for T2DM.

## 2. Methods

The protocol for this systematic review is registered at http://www.crd.york.ac.uk/PROSPERO/display_record.asp?ID=CRD42017056848.

### 2.1. Search Strategy

We searched the databases Medline, Embase, CINAHL and the Cochrane library from their inception until March 2018. Searches were limited to the English language. ClinicalTrials.gov and the World Health Organization International Clinical Trials Registry Portal were searched for ongoing studies. Reference lists of included studies were checked for additional studies, but none were identified. See Supplementary Table S1 for an example database search strategy. 

### 2.2. Selection Criteria

#### 2.2.1. Inclusion Criteria

Population: women with gestational diabetes mellitus.

Study design: any prospective or retrospective primary study, including qualitative studies (interviews or surveys), controlled trials and observational studies, or systematic reviews of:

- Barriers to postnatal screening for T2DM in women with GDM. All study types included.
- Interventions: any intervention to improve uptake of postnatal screening for T2DM screening versus any other intervention or no intervention.

Setting: UK NHS or countries with similar healthcare systems. These were defined as countries with most care provided by general practitioners (GPs) rather than women receiving primary care in the community from specialists, and included, but were not limited to, Australia, Belgium, Canada, Ireland and France.

#### 2.2.2. Exclusion Criteria

We excluded studies of predictors of T2DM and interventions and barriers to lifestyle changes to prevent later T2DM. We also excluded primary studies and reviews of different tests for GDM, unless uptake was reported as an outcome. Owing to resource constraints, studies not in the English language were excluded.

### 2.3. Study Selection

Screening of titles and abstracts was undertaken independently by two reviewers and full text manuscripts obtained for further scrutiny. In the event of any disagreement, a third reviewer was involved to make a final decision. The inclusion criteria were then applied to the retrieved manuscripts by the two reviewers and an agreement reached on final inclusions. 

### 2.4. Data Extraction

One reviewer used a pre-defined data extraction form to extract data from the included studies. Data extractions were checked by a second reviewer. All discrepancies were resolved through discussion.

### 2.5. Quality Assessment

We used the Critical Appraisal Skills Programme checklist [20] for qualitative studies (and a small number of other studies where we considered this the most appropriate tool). For all other manuscripts, the National Institute of Health quality assessment tools [21] were used. One reviewer quality assessed the included studies, and this was checked by a second reviewer during checking of the data extraction. Any discrepancies were resolved through discussion or with the view of a third reviewer.

### 2.6. Data Synthesis and Summary Measures

As this systematic review includes a variety of study designs, including qualitative studies, meta-analysis of the results was not feasible. A narrative synthesis of the results is therefore presented. We have categorised studies into several themes to aid interpretation of their results. The data extractions were used to identify, extract and compare key themes from each paper. This allowed similarities and contradictions to be identified across the studies, enabling one author to categorise themes from the tabulated summaries. These themes were then reviewed by co-authors to determine the final appropriate themes.

## 3. Results

**Figure 1:**
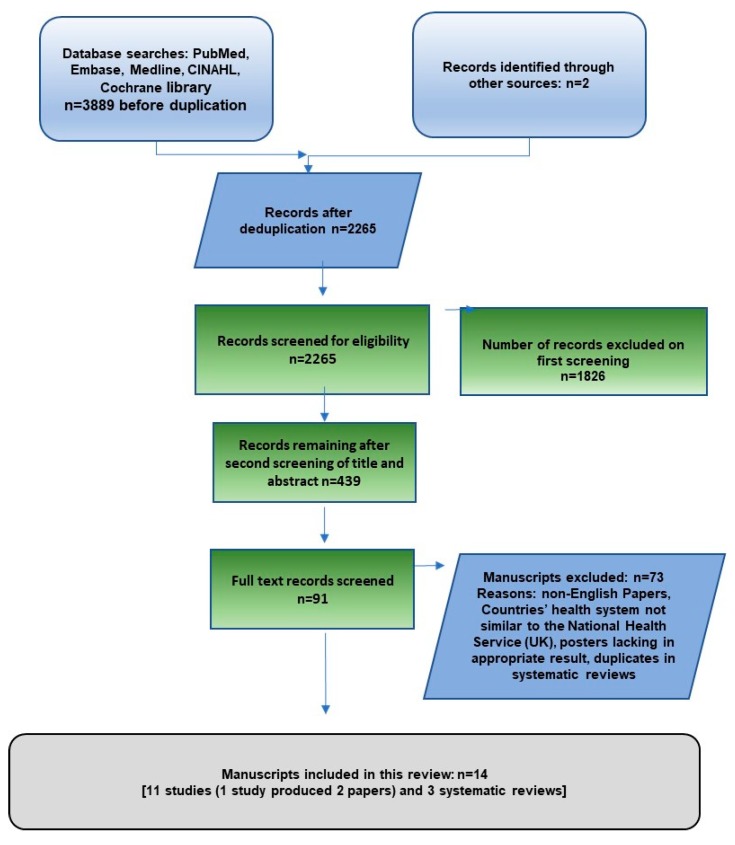
Flow-chart of the identification of included studies and systematic reviews.

This systematic review is reported in accordance with the PRISMA statement [22]. After screening 2265 records, 91 full text papers were retrieved. The titles and abstracts of these were examined against the eligibility criteria by two independent reviewers. Eleven primary studies and three systematic reviews were included (Figure 1). These systematic reviews included a large number of studies, (58 [9], 6 [23] and 42 [24] studies, respectively) many of which did not meet our inclusion criteria. A number of studies were not relevant to an NHS setting and not all studies were in the postnatal setting. Included primary studies in this review were qualitative/interview studies (*n* = 2), RCTs (*n* = 1), before and after studies (*n* = 2) and cohort studies (*n* = 6). They cover several countries including the UK, Canada and Australia, and were published from 2003 to 2017. Table 1 describes the characteristics of the included primary studies and systematic reviews. 

### 3.1. Quality Assessment

Of the 11 primary studies, four were rated good, six were rated fair and one poor (Supplementary Table S2). Of the three systematic reviews, one was rated good, one fair and one poor. Some of the primary studies were rated with lower quality due to lack of information on their methods, rather than because of a known risk of bias.

### 3.2. Identified Themes

There were seven themes identified in the included primary studies and systematic reviews, although there was some overlap between these: the OGTT itself; demands on maternal time; education and information; risk perception; professional knowledge; coordination of care and reminder systems. The results of these themes were considered for both facilitators and barriers to screening. Table 1 shows the results for each study/systematic review and the themes they were matched to. 

Five primary studies [25,26,27,28,29] and 2 systematic reviews [9,24] reported on the barriers that would reduce the likelihood of a woman attending postnatal screening for T2DM, which included the OGTT itself, demands on maternal time, lack of knowledge, risk perception and knowledge of the relevant health professionals. Suggestions to increase uptake were reported in 10 primary studies [25,26,27,28,29,30,31,32,33,34,35] and 2 systematic reviews [9,23]. The key suggestion that was reported by 8 primary studies [26,27,28,29,30,31,32,33,35] and 1 systematic review [23] was the use of reminder systems. Other suggestions included effective education and information and a shorter test duration.

#### 3.2.1. OGTT

As discussed above, the OGTT is the recommended test to diagnose T2DM, however, several primary studies described the OGTT as a barrier [24,25,26,27], for example not fitting in with childcare, being unable to arrange transport to take the test, too soon after an operative birth and being too busy with the new baby. In a study by Sterne et al. [27], 36% of women said the OGTT was inconvenient and too time consuming. In the same study, 10% of women described the test as unpleasant due to the taste, the long fast and the fear of needles. Women with GDM expressed their desire for a more palatable quicker test [24]. Although not described formally as a barrier, Benhalima et al. [30] stated that the fasting plasma glucose test was used rather than OGTT because it was more practical and achievable.

Van Ryswyk et al. [29] showed that of 165 women who completed their OGTT, 86% found the test easy to fast for; 63% did not have any difficulty finding the time; 78% were happy with the test experience. However, 33% of women said a shorter test would have facilitated attendance. Sterne et al. [27] concurred, reporting that 16% of women in their study believed that a more convenient and shorter test would improve uptake. Minsart et al. [26] also found women would prefer a faster test. Van Ryswyk et al. [29] expands on this with women desiring suitable baby friendly facilities at testing sites and developing home testing service along with combining tests with other postnatal checks.

#### 3.2.2. Demands on Maternal Time

During pregnancy, women were focused on protecting their unborn child and would abide by all advice and expressed guilt around how their actions could detrimentally affect their child’s health [25]. However, once the baby was born, all attention turned to caring for the child and the mother saw her own health as secondary to the needs of the child. Maternal duties and prioritization of the new baby become a barrier to the women engaging in postnatal screening. Van Ryswyk et al. [29] found that 73% of women stated lack of time while 30% had trouble with childcare and wanted to focus on the newborns’ needs as barriers to uptake of the test. Nielsen et al. [28] found that women thought follow-up screening was of high importance but the commitments of everyday life and that of a new baby reduced the priority of screening and therefore affected participation rates. 

#### 3.2.3. Education and Information

Women described postnatal education and information as vague and inadequate. Minsart et al. [26] described how some women thought that because they had implemented lifestyle changes antenatally they were no longer at risk of T2DM and that postnatal tests were unnecessary. Sterne et al. [27] found lack of awareness around the need for postnatal screening to be a barrier in 28% of women with GDM. This was considered to be linked to poor clinician communication both in the antenatal and postnatal period, although a few (6%) simply forgot about the test. The study also found that women who had an awareness of the need for postnatal screening for T2DM, either through effective health professional communication or due to a family history of T2DM, were 31% more likely to have screening. Another study found some women did not understand the need for the test; some continued to home test their glucose levels and did not think they needed the test; other women became pregnant again or did not want to take the test [24,29]. Nielsen et al. [28] found that women were aware of the risk of T2DM and the need for postnatal screening but cited the lack of time during appointments to ask questions and gain further information. Most women were given leaflets, but little clarification or open discussion was available. 

In a set of interviews with 13 women with GDM in Australia, it was suggested that when women found that their information needs (e.g., why follow-up screening was necessary) were met by clinicians, their experiences were described more positively, and they were found to be more likely to undertake postnatal screening [25]. Cosson et al. [34] evaluated the French IMPACT information and incentive campaign that targeted women with GDM and provided community multidisciplinary education and information to the women and their primary caregivers. This intervention significantly increased the uptake of OGTT from 43.1% to 57.5% (*p* < 0.001).

#### 3.2.4. Risk Perception and Fear 

Women viewed GDM as a condition of pregnancy, and thought that once the baby was born, they were no longer at risk. Some women stated that they had been reassured by health care professionals that this was the case. Kilgour et al. [25] described how GDM was very much talked about during the pregnancy but the minute the baby was born it was not mentioned again other than to say it was no longer a problem.

Kilgour et al. [25] similarly found that if the condition was described as not a high priority but only as a mild condition of pregnancy, women were less likely to undertake postnatal screening. Women said that they should have been told about the importance of postnatal screening at the time of diagnosis and this would have increased their perception of risk and likelihood of taking up the screening. 

Minsart et al. [26] found that women were not only unaware of the postnatal risks to themselves but also to their children: 8% of mothers recalled being told of the risks of T2DM and the increased risk of obesity for their children. The study also found that women who had undergone postnatal screening were more likely to have a moderate to high-risk perception of developing T2DM than a no or a low risk perception (81.8% versus 18.2%).

Van Ryswyk et al. [30] found that 15% of women with GDM did not believe that they were at high risk of T2DM, and so did not have the test, although another 15% who were concerned they were at high risk also did not have the test. Conversely, Sterne et al. [26] found that only 1% of women with GDM stated fear as a barrier to screening, whilst 15% of women who had had GDM found fear of T2DM increased uptake of postnatal screening. 

It appears from the included studies that information needs to be consistent throughout the pregnancy into the postnatal period. Inconsistent information can alter perceptions of risk with postnatal screening for T2DM no longer seen as important. 

#### 3.2.5. Professional Knowledge/Continuity and Coordination of Care

Minsart et al. [26] found women described a dramatic difference in care in the postnatal period. Antenatally, they received intensive care but felt that they were left alone in the postnatal period [24]. Women felt that their unborn baby was the focus of antenatal appointments, and their individual circumstances were of little importance; they felt that they were not actively involved in their own care and felt detached from decision-making, which gave rise to feelings of anxiety and an inability to communicate to health providers [28]. Furthermore, women found that their GPs had a lack of knowledge of their risks and the need for screening, and women found that there were no reminders to have checks after the baby was born [9].

Kilgour et al. [25] described one woman who had repeatedly sought information and education about her condition but had been repeatedly told not to worry, so by the time it came to her postnatal screening test for T2DM she was not worried and did not have the test.

Kilgour et al. [25] also found that the emphasis health professionals put on postnatal screening during the antenatal period and how they described the seriousness of the condition influenced women positively to undertake postnatal screening.

Lack of coordination between secondary and primary care was described as a barrier. Nielsen et al. [28] reported women describing their antenatal and postnatal care as fragmented and lacking in continuity and coordination between departments and health professionals. Women thought that postnatal screening appointments were left to them to remember and arrange, and that some GPs were unaware of the need for screening which could potentially affect the women’s decision to have screening. Some women stated that GDM and the associated long-term health complications were not discussed at all at their follow-up appointments.

In Canada, Clark et al. [13] found that women with GDM did not have postnatal screening for T2DM with the OGTT as per the 1998 Canadian Diabetes Association Guidelines. Instead, women with GDM had either serum glucose or glycated haemoglobin measurements. This could reflect either a lack of knowledge on the part of the clinicians delivering the screening recommendations or a preference of women for serum glucose or glycated haemoglobin testing. These tests could be more convenient for the patient when compared to the OGTT. 

Being under the care of an endocrinologist insignificantly increased the likelihood of postpartum screening among less educated Australian women and being seen by a diabetes educator significantly increased the likelihood of postpartum screening among Australian women under the care of an obstetrician [9].

#### 3.2.6. Reminder Systems

Reminder systems were the most frequently suggested intervention to improve screening. In a large Belgian study, 5465 women were included in a register which sent automated letters and emails 3 months after delivery and then subsequent reminders for up to 6 years to those who had not yet undertaken the screening test [30]. All the women had voluntarily enrolled in the register after a diagnosis of GDM, and response rates for the screening tests postpartum ranged from 67.4% after the first year to 71.9% after the fifth year. In a survey, 70.5% of women indicated that the reminders were helpful, and they would have been unlikely to attend without them. The authors state that the efficacy of the recalls was demonstrated, although it is noteworthy that the fasting plasma glucose test was used rather than the OGTT, and that there was a low response rate to the survey of women (26.4%). 

Nielsen et al. [28] reported that women stated that a reminder by e-mail, letter, phone or laboratory request form would facilitate screening test uptake, and that this would also relieve anxiety. Sterne et al. [27] reported that 39% of women thought a reminder system would be beneficial, however, Minsart et al. [26] noted that despite this, women did not always act on reminders, with only 44% undertaking postnatal screening for T2DM despite receiving one.

In their systematic review, Nielsen et al. [9] identified four studies that found the use of reminders had positive results. Limited details of the reminders were reported in the review, except that for one of these four studies, from Australia, written information or individualised risk reduction advice were given, and these both significantly increased the likelihood of screening. In a separate study, Carmody et al. [31] examined the effect of a reminder letter and phone call by a central coordinator on uptake rates of postnatal OGTT. Following the appointment of the coordinator, OGTT rates increased by 12% from the previous year, and this increase was sustained over the following 4 years. The intervention had an overall success rate with 75.6% of women with GDM having the test over 5 years. 

Halperin et al. [32] conducted a quality improvement initiative where patients were sent e-mail reminders and GPs were sent fax reminders 1 month before women were due their postnatal screening appointment. This increased screening rates from 33% to 44% over the 18-month intervention. Alongside these reminder interventions, obstetric summaries were improved to ensure that GPs were getting the information about their patients’ GDM diagnosis, and the need for them to have postnatal screening for T2DM. Despite the improvements, the results were far below the desired 60% the programme was aiming to achieve.

Combining education with reminders was a successful strategy in one study [33], in which women who had both education and reminders were 2–3 times more likely to have an OGTT within the first year after delivery. Women who were given the education but not sent reminders were less likely to attend for an OGTT (19.2%) than those who were sent reminders (between 31.2% and 45.9% across two sites).

Van Ryswyk et al. [29,35] reported a RCT that evaluated the use of [short message service (SMS) or text message] reminders for postnatal screening for T2DM at six weeks postpartum. There was no increase in the uptake of OGTT by six months postpartum but there were high rates of uptake in both the reminder group and control group of 77%. However, >88% of the women had received a postal reminder from the national reminder scheme and discharge summaries including patient’s GDM diagnosis and the need for a postnatal OGTT were sent to 98% of the women’s GPs in both groups. These additional reminders along with the participation bias could have affected both groups but could also suggest that the combination of these aspects could increase uptake rates to a high level. The follow-up questionnaire after the RCT found that 70% of women preferred a reminder by SMS, 17% by e-mail 12% by post and <1% by telephone call [29,35].

A systematic review specifically of reminders for women with GDM or their health care professionals by Jeppesen et al. [23] included six primary studies. The types of reminders for women varied from postal, email and phone messages, and the review found that the effects of these reminders varied between the included studies. Patient reminders with a personal approach such as the phone calls or personal letters could have a larger effect in persuading women to attend postnatal screening. In addition, the number of reminders influenced the rate of uptake of screening tests. The review also found that it is plausible that reminders to healthcare providers could increase postnatal follow-up [23]. However, this was based on the findings from two included studies only. 

## 4. Discussion

Attending postnatal screening for continuing hyperglycaemia is important for women who have had GDM. Once these women have been identified through screening, they can be offered support and appropriate treatment to prevent the development of T2DM, or to treat it. Studies have looked at both facilitators and barriers to try to determine why screening uptake rates are so low. The focus of this systematic review was to identify how the UK NHS can increase postnatal screening rates. From the studies we reviewed, the barriers to better uptake of postnatal testing include:

- The OGTT test.
- Competing demands on maternal time.
- A lack of education and information.
- Risk perception and fear.
- Knowledge amongst health care professionals.
- Problems with continuity and coordination of care, e.g., poor communication between professionals, including from secondary to primary care.

Interventions to improve uptake include:

- Reminders—the intervention with the largest evidence base.
- Increasing awareness of GDM and the risk of subsequent T2DM, by education.
- A more user-friendly and convenient blood glucose test than the OGTT.

Postnatal screening rates for T2DM are suboptimal in several countries. However, the Carmody et al. (Ireland) and Van Ryswyk et al. (Australia) studies have all shown good uptake rates of over 70% [29,31].

Reminder systems were the commonest intervention to increase uptake but were heterogeneous and had mixed results. Adding a personal touch to the reminder appears to help [23]. It is the intervention most suggested by women when asked what would increase uptake. An intervention that addresses other barriers such as awareness of personal risk or education in combination with a reminder might be more successful [9,33].

Improved communication of risk by professionals as a facilitator might also help. Some women reported that communication from their healthcare providers was poor [27]. Clinicians tended to reassure women that their condition was limited to pregnancy and did not highlight the risks of T2DM. Educational interventions postnatally could address illness perceptions in women with GDM.

Women thought that the diagnosis of GDM was a prime time for reinforcing the need for postnatal screening for hyperglycaemia, but this opportunity was often missed [25]. Women thought that antenatal appointments were good opportunities to understand their condition, gain knowledge, participate in their own care and reinforce the need for postnatal screening [24,25,26,27]. Many women had little understanding of their condition, potential future risks or how they could reduce these risks [24,26,27]. Pennington et al. [36] studied the barriers and enablers to postnatal care and found risk denial, apathy in changing lifestyle, poor health literacy and lack of social support as barriers to follow-up care. They also identified differences in the quality of care provided by GPs, identifying a lack of awareness, knowledge and no guidelines as barriers to care.

The OGTT was named as a barrier by some women, citing the length of time needed for the test and the unpleasantness of the test as reasons for poor uptake [24,25,26,27]. However, the number of women who found little problem with the test outnumbered those that did [27,29]. 

The greatest problem for mothers was trying to fit in the test with the needs of their newborn. Suggestions to improve this included home testing (for example with the health visitor), combining the test with other postnatal visits and making the sites more baby friendly.

Another issue is the coordination of postnatal follow-up. In the past, this was done in the hospital environment by the obstetrics team. However, it is becoming more common in the UK for postnatal care to be done in primary care. Pierce et al. [37] carried out a national postal survey with questionnaires sent to a selection of GPs and an obstetrician and diabetologist in every hospital; the results also suggests that only 39% of GPs had protocols for the management of women who had had GDM and only one third of these had been agreed with local secondary care services: 19% of GPs were not being informed that their patients had a diagnosis of GDM. The results also showed that OGTT was not the primary test being used and women were not consistently having their follow-up in primary care. Lack of coordination and knowledge may be due to healthcare professionals being unsure who is responsible for postnatal follow-ups. The results also suggest that NICE guidance is not being followed. Similarly, in Canada, the Canadian guidance was not followed in the care of women with GDM [13]. Therefore, high-quality continuity and coordination of care with good communication between professionals would improve postnatal screening.

The risk of developing T2DM can continue into the future. Improving postnatal uptake should be beneficial in the short term but the effect on screening over the years that follow is uncertain. 

### Strengths and Limitations 

Strengths of this systematic review include the rigorous methodology. Both qualitative and quantitative data were used, and the variety of study designs allows for a full picture of the barriers and facilitators to women’s uptake of screening within the context of the UK NHS. The PRISMA statement (Supplementary Table S3) was used as a guide to ensure a high-quality systematic review. 

Our systematic review updates evidence from previous systematic reviews in the area, including new evidence since these existing reviews were published. Our review was focused on studies relevant to the UK NHS. On the other hand, this limits the generalizability of our findings to other healthcare systems that are not similar to the UK NHS. 

Limitations include the quality of a large proportion of the included studies being assessed as only fair or poor (although some studies are rated fair due to the lack of reporting of methods and may be good quality). Publication bias was not examined, and we were unable to include studies published in non-English languages. While the populations within the individual studies varied, with some with only small samples, the review covers more than 13,500 women in total. Loss to follow-up varied from minimal to high; this was considered within the quality assessments.

## 5. Conclusions

Our review has focused on studies relevant to the UK NHS and has identified a number of barriers to the uptake of screening after GDM. These include competing demands on maternal time, issues around the test itself, and limited appreciation of the need for testing. Consideration needs to be given to these barriers in the antenatal and postnatal periods, and before subsequent pregnancies. Interventions that aim to increase uptake include reminder systems, and studies suggest these may improve postnatal screening rates, especially if they are combined with education. However, the heterogeneity of the studies and their interventions mean we can only make tentative conclusions. 

### Implications for Future Research

Current recommendations state that testing should be done using an OGTT, but this is time consuming and inconvenient, and is one of the cited reasons women are not having postnatal follow-up screening. Research is needed to establish if there is a quicker test to identify continuing hyperglycaemia, and one possibility is testing before discharge, since glucose tolerance returns to normal soon after delivery [38].

## Figures and Tables

**Table 1:** Characteristics of included primary studies and systematic reviews (*n* = 14).

Author (Year) Country Study Design Quality	Methods of Ascertainment of Barriers/Description of Intervention	Participants (Women with GDM)	Key Findings	Themes
*Studies addressing barriers to the uptake of postnatal screening for T2DM*				
Kilgour (2015) Australia. Qualitative study. Good quality.	Convergent interviews by experienced nurse and midwife, broad questions and prompts to ensure research aims addressed, approximately 40 min.	13	All 13 women knew of the need for a postpartum follow-up however only 7 undertook the screening. The main theme was need for information (including diagnosis of GDM; seeking GDM information; accessing specialist services; the need for postnatal screening follow-up and completing the post-natal GDM follow-up).	Risk perception; education and information; coordination of care.
Minsart (2014) Belgium. Observational cross-sectional survey. Fair quality.	Telephone survey about risk perception of diabetes, and reasons for avoiding screening and strategies that would have persuaded them to undertake the test.	72	Reasons for not attending the postpartum test included: unaware of the necessity (50%); insufficient information (42.9%); lack of time (32.1%); OGTT not convenient (17.9%); fear of diagnosis (7.2%); did not believe they were at risk anymore (3.6%); thought not necessary (25%).	Reminder systems; risk perception; education and information; coordination of care; OGTT.
Nielsen (2015) Denmark. Qualitative study. Good quality.	Semi-structured interviews undertaken by one researcher at woman’s home, diabetes clinic or researcher’s office. A 4-step analytical approach used to identify meaningful units and abstract and summarize content.	7 (40 invited).	Four key themes for non-attendance: fragmented care; insufficient information; focus on physiological aspects of birth and health of baby; risk perception in everyday life.	Reminder systems; risk perception; education and information; coordination of care.
Sterne (2011) Australia. Observational cross-sectional interview survey/qualitative. Fair quality.	Telephone interview, approximately 10 min, standard interview form and recorded via audiotape. Questions included demographic and clinical questions and open-ended questions about barriers and facilitators to attending screening. Tick list of common barriers used. Prompts provided for potential facilitators.	88 (187 eligible).	Barriers included lack of awareness; forgetting; inconvenience of the test; dislike of the drink for the test; fear of diabetes (also a facilitator in some).	Reminder systems; risk perception; education and information; OGTT.
*Systematic reviews addressing barriers to the uptake of postnatal screening for T2DM*				
Nielsen (2014) 19 USA, 9 Australia, 6 Canada, 1 Denmark, 1 Netherlands. Poor quality.	58 studies included; 36 focused on postpartum follow-up; of these 5 studies addressed barriers to uptake of postpartum screening. Two studies assessed women with GDM via a survey and qualitative study respectively (3 reported perceptions of health care providers).	344	Barriers from women included time pressure, lost requisition, adjustment to new baby, baby’s health issues, delivery experience, feeling healthy and not in need of follow-up or fear of bad news, and experiences with medical care and services.	Reminder systems; risk perception; professional knowledge; coordination of care; demands on maternal time.
Van Ryswyk (2015) 12 USA, 10 Australia, 7 Canada, 3 United Kingdom, 3 Sweden, 1 Denmark, 1 France, 1 Austria, 2 Brazil, 1 Tonga, 1 Vietnam. Good quality.	42 studies included that were qualitative or survey studies assessing barriers, facilitators and attitudes to postnatal care and follow-up including towards postpartum blood testing and use of reminders for follow-up.	7949	Barriers to postpartum screening included; lack of understanding around the importance; not seeing the need; forgetting; another pregnancy; lost laboratory forms; not wanting to take the test; not liking the test experience, demands on maternal time.	Reminder systems; risk perception; education and information; coordination of care; demands on maternal time; OGTT.
*Studies addressing barriers to the uptake of postnatal screening for T2DM and interventions to increase uptake*				
Van Ryswyk (2015) Van Ryswyk (2016) Australia. Cross-sectional survey and RCT. Good quality.	Intervention: text reminder to attend OGTT at 6 weeks postpartum, and further reminders at 3 and 6 months if required. Control group received 1 text reminder at 6 months. Barriers: Women sent a questionnaire that could be completed via email, post or over the telephone.	Van Ryswyk (2015)—276 (140 intervention and 136 control). Van Ryswyk (2016)—275 sent questionnaire, 208 completed.	The intervention group did not increase attendance for OGTT within 6 months postpartum compared with the control group [77.6% versus 76.8%, relative risk 1.01 (95% CI 0.89, 1.15)]. The most frequently cited barriers for non-attendance: not having enough time (73%), childcare (30%); need to focus on baby (30%); test too long (18%), perceived low risk type 2 diabetes (15%), anxiety of being diagnosed with type 2 diabetes (15%).	Reminder systems; risk perception; coordination of care; demands on maternal time; OGTT.
*Studies of interventions to increase uptake of postnatal screening for T2DM*				
Benhalima (2017) Belgium. Observational cohort study and survey. Good quality.	Automated recall via a letter and email 3 months after delivery to determine whether testing was made at 6–12 post-partum screening, where appropriate reminder letters and emails after 11 months then yearly with advice to visit GP for FPG test. Non-responders received an email/telephone call, or SMS reminder.	5465 (500 in the survey).	58.8% had a postpartum screening test; 2.8% of these reported having diabetes. Yearly response rates varied from 74.4% after the first year to 61.8% after the fifth year. The proportion reporting a screening test varied from 67.4% after the first year to 71.9% after the fifth year.	Reminder systems.
Carmody (2015) Ireland. Observational (prospective cohort). Fair quality.	Central coordinator used to facilitate attendance. Contact was written and verbal. Postdelivery women were verbally reminded of the need for a postpartum follow-up. Post 2009 a postal reminder and a telephone call was used.	1520	75.6% had a postpartum OGTT. After appointment of the coordinator in 2009 there was a 12% increase in attendance on the previous year. There was a significant difference between attendance rates in 2008 compared with subsequent years (69.0% versus 77.7%, *p* ≤ 0.001	Reminder systems.
Clark (2003) Canada. Before and after study. Fair quality.	Introduction of the CDA guideline recommending that all women with a diagnosis of gestational diabetes be screened postpartum for type 2 diabetes (OGTT 6 weeks to 6 months postpartum).	254 (131 before and 123 after).	No women had an OGTT either before or after the guidance. 72.5% had a serum glucose in 1997 compared to 92.3% in 2000 (difference 20%, *p* < 0.05).	Coordination of care; OGTT.
Cosson (2015) France. Before and after study. Fair quality.	IMPACT initiative (health advice, care giver reminder letters)	961 (589 before and 372 after).	The postpartum screening rate during the first 6 months postpartum was greater after (48.9%) the IMPACT campaign than before (33.3%), OR 1.7 (95% CI 1.1–2.5)	Education and information; coordination of care.
Halperin (2015) Canada. Before and after study. Fair quality.	1. Improvements in physicians’ dictations 2. Patient-directed e-mail reminder systems 3. Family physician directed fax reminder systems	300	44% had an OGTT within 6 months; an 11% increase to the 18 months prior to the intervention, *p* = 0.008. Results increased to 50% by 12 months postpartum (not significant from 42% baseline).	Reminder systems; coordination of care.
Peticca (2014) Canada. Observational study (retrospective cohort with intervention and no intervention). Poor quality.	A reminder package was posted within 3 months of delivery to women who attended diabetes education classes at 2 of 3 sites. Women who attended the third site received no reminder but were given education on the importance of a postpartum follow-up appointment with screening.	546 (338 with intervention and 208 with no intervention).	Rates of OGTT completion at 12 months was 38% in those who attended clinics with reminders and 19% in those who attended clinics without reminders (*p* < 0.001).	Reminder systems
*Systematic reviews of interventions to increase uptake of postnatal screening for T2DM*				
Jeppesen (2015) 3 Canada, 1 USA, 1 Australia, 1 Finland. Fair quality.	6 studies included; Reminder interventions to patients (postal, email, phone/text messages) or health professionals (also including pop-up electronic reminders and alerts or notes on medical reports) were eligible.	1261	All six studies included reminder systems for patients: two studies showed benefits of phone call reminders (28% attendance for OGTT versus 13.75% in control site in one study; 83.2% who completed an OGTT had received a phone reminder compared with 49.1% who did not). The number of reminders influenced the completion rate of screening tests. 80% after the first reminder, 41% of the remaining after the second reminder, and after the third reminder 28% completed the test in one study. In another study up to six reminders were sent over a 6-year period; 56.3% of women at the first reminder and 66.7% after the sixth reminder reported having an OGTT (numbers declined over time however).	Reminder systems; professional knowledge; coordination of care.

CDA: Canada Diabetes Association; CI: confidence interval; FPG: fasting plasma glucose; GDM: gestational diabetes mellitus; GP: general practitioner; OGTT: oral glucose tolerance test; OR: odds ratio; RCT: randomised controlled trial; SMS: short message service; T2DM: type 2 diabetes mellitus; USA: United States of America.

## Supplementary Materials

The following are available online at http://www.mdpi.com/2077-0383/8/1/4/s1, Table S1: Example database search strategy, Table S2: Quality assessments, Table S3: PRISMA Checklist.
