# Supplementary material for: Improving Uptake of Postnatal Checking of Blood Glucose in Women Who Had Gestational Diabetes Mellitus in Universal Healthcare Settings: A Systematic Review
Source: J Clin Med. 2018 Dec 20;8(1):4. doi: 10.3390/jcm8010004 (PMC6352125; doi:10.3390/jcm8010004)
Supplement: Supplementary file 1 [file jcm-08-00004-s001.zip › Table S1.pdf]

Example database search strategy; the search strategy below was used for the Ovid Medline search and then adapted as appropriate to the other databases.

	**Search term**
1	exp Diabetes, Gestational/
2	gestational diabetes.tw.
3	1 or 2
4	exp Postpartum Period/
5	(postpartum or postnatal or post-partum or post-natal or post-delivery or postdelivery or post-birth or postbirth).tw.
6	4 or 5
7	3 and 6
8	(uptake or non-uptake or detect* or management or follow-up or recurren* or assess* or identif* or screen* or test* or barrier* or recall or attend* or adhere* or complian* or comply or participat*).tw.
9	exp Patient Compliance/
10	8 or 9
11	7 and 10
All databases were searched from their inception (1946 for Medline, 1974 for Embase, 1979 for CINAHL, 1993 for Cochrane Library) to the 19th March 2018.	
