# Supplementary material for: Improving Uptake of Postnatal Checking of Blood Glucose in Women Who Had Gestational Diabetes Mellitus in Universal Healthcare Settings: A Systematic Review
Source: J Clin Med. 2018 Dec 20;8(1):4. doi: 10.3390/jcm8010004 (PMC6352125; doi:10.3390/jcm8010004)
Supplement: Supplementary file 1 [file jcm-08-00004-s001.zip › Table S2.pdf]

Quality assessments

Benhalima (2017)

Criteria	Yes	No	Other (CD, NR, NA)*
1. Was the research question or objective in this paper clearly stated?	Yes		
2. Was the study population clearly specified and defined?	Yes		
3. Was the participation rate of eligible persons at least 50%?			CD
4. Were all the subjects selected or recruited from the same or similar populations (including the same time period)? Were inclusion and exclusion criteria for being in the study prespecified and applied uniformly to all participants?	Yes		
5. Was a sample size justification, power description, or variance and effect estimates provided?			NA
6. For the analyses in this paper, were the exposure(s) of interest measured prior to the outcome(s) being measured?			NA
7. Was the timeframe sufficient so that one could reasonably expect to see an association between exposure and outcome if it existed?	Yes		
8. For exposures that can vary in amount or level, did the study examine different levels of the exposure as related to the outcome (e.g., categories of exposure, or exposure measured as continuous variable)?			NA
9. Were the exposure measures (independent variables) clearly defined, valid, reliable, and implemented consistently across all study participants?	Yes		
10. Was the exposure(s) assessed more than once over time?	Yes		
11. Were the outcome measures (dependent variables) clearly defined, valid, reliable, and implemented consistently across all study participants?			CD
12. Were the outcome assessors blinded to the exposure status of participants?			NA
13. Was loss to follow-up after baseline 20% or less?		No	
14. Were key potential confounding variables measured and adjusted statistically for their impact on the relationship between exposure(s) and outcome(s)?		No	
**Quality Rating (Good, Fair, or Poor) (see guidance)**			
Rater #1 JC: Good			
Rater #2 EL: Good			
Additional Comments (If POOR, please state why):			

***CD, cannot determine; NA, not applicable.**

Carmody (2015)

Criteria	Yes	No	Other (CD, NR, NA)*
1. Was the research question or objective in this paper clearly stated?	Yes		
2. Was the study population clearly specified and defined?	Yes		
3. Was the participation rate of eligible persons at least 50%?			NR
4. Were all the subjects selected or recruited from the same or similar populations (including the same time period)? Were inclusion and exclusion criteria for being in the study prespecified and applied uniformly to all participants?		No	
5. Was a sample size justification, power description, or variance and effect estimates provided?		No	
6. For the analyses in this paper, were the exposure(s) of interest measured prior to the outcome(s) being measured?			NA
7. Was the timeframe sufficient so that one could reasonably expect to see an association between exposure and outcome if it existed?	Yes		
8. For exposures that can vary in amount or level, did the study examine different levels of the exposure as related to the outcome (e.g., categories of exposure, or exposure measured as continuous variable)?			NA
9. Were the exposure measures (independent variables) clearly defined, valid, reliable, and implemented consistently across all study participants?	Yes		
10. Was the exposure(s) assessed more than once over time?			NA
11. Were the outcome measures (dependent variables) clearly defined, valid, reliable, and implemented consistently across all study participants?	yes		
12. Were the outcome assessors blinded to the exposure status of participants?		No	
13. Was loss to follow-up after baseline 20% or less?			NR
14. Were key potential confounding variables measured and adjusted statistically for their impact on the relationship between exposure(s) and outcome(s)?	Yes, measured but not adjusted.		
**Quality Rating (Good, Fair, or Poor) (see guidance)**			
Rater #1 HS: Fair			
Rater #2 JC: Fair			
Additional Comments (If POOR, please state why):			

***CD, cannot determine; NR, not reported; NA, not applicable.**

Clark (2003)

**Criteria**	**Yes**	**No**	**Other (CD, NR, NA)***
1. Was the study question or objective clearly stated?	Yes		
2. Were eligibility/selection criteria for the study population prespecified and clearly described?	Yes		
3. Were the participants in the study representative of those who would be eligible for the test/service/intervention in the general or clinical population of interest?	Yes		
4. Were all eligible participants that met the prespecified entry criteria enrolled?		No	
5. Was the sample size sufficiently large to provide confidence in the findings?	Yes		
6. Was the test/service/intervention clearly described and delivered consistently across the study population?		No	
7. Were the outcome measures prespecified, clearly defined, valid, reliable, and assessed consistently across all study participants?	Yes		
8. Were the people assessing the outcomes blinded to the participants' exposures/interventions?		No	
9. Was the loss to follow-up after baseline 20% or less? Were those lost to follow-up accounted for in the analysis?			NA
10. Did the statistical methods examine changes in outcome measures from before to after the intervention? Were statistical tests done that provided p values for the pre-to-post changes?	Yes		
11. Were outcome measures of interest taken multiple times before the intervention and multiple times after the intervention (i.e., did they use an interrupted time-series design)?			NA
12. If the intervention was conducted at a group level (e.g., a whole hospital, a community, etc.) did the statistical analysis take into account the use of individual-level data to determine effects at the group level?			NA
**Quality Rating (Good, Fair, or Poor) (see guidance)**			
Rater #1 HS: Fair			
Rater #2 EL: Fair			
Additional Comments (If POOR, please state why):			

***CD, cannot determine; NR, not reported; NA, not applicable.**

Cosson (2015)

Criteria	Yes	No	Other (CD, NR, NA)*
1. Was the research question or objective in this paper clearly stated?	Yes		
2. Was the study population clearly specified and defined?	Yes		
3. Was the participation rate of eligible persons at least 50%?	Yes		
4a. Were all the subjects selected or recruited from the same or similar populations (including the same time period)?<br>4b. Were inclusion and exclusion criteria for being in the study prespecified and applied uniformly to all participants?		No<br>No	
5. Was a sample size justification, power description, or variance and effect estimates provided?	Yes		
6. For the analyses in this paper, were the exposure(s) of interest measured prior to the outcome(s) being measured?			NA
7. Was the timeframe sufficient so that one could reasonably expect to see an association between exposure and outcome if it existed?	Yes		
8. For exposures that can vary in amount or level, did the study examine different levels of the exposure as related to the outcome (e.g., categories of exposure, or exposure measured as continuous variable)?			NA
9. Were the exposure measures (independent variables) clearly defined, valid, reliable, and implemented consistently across all study participants?	Yes		
10. Was the exposure(s) assessed more than once over time?			NA
11. Were the outcome measures (dependent variables) clearly defined, valid, reliable, and implemented consistently across all study participants?	Yes		
12. Were the outcome assessors blinded to the exposure status of participants?		No	
13. Was loss to follow-up after baseline 20% or less?		No	
14. Were (a) key potential confounding variables measured and (b) adjusted statistically for their impact on the relationship between exposure(s) and outcome(s)?	Yes		
**Quality Rating (Good, Fair, or Poor) (see guidance)**			
Rater #1 HS: Fair			
Rater #2 JC: Fair			
Additional Comments (If POOR, please state why):			

***CD, cannot determine; NR, not reported; NA, not applicable.**

Halperin (2015)

**Criteria**	**Yes**	**No**	**Other (CD, NR, NA)***
1. Was the study question or objective clearly stated?	Yes		
2. Were eligibility/selection criteria for the study population prespecified and clearly described?		No	
3. Were the participants in the study representative of those who would be eligible for the test/service/intervention in the general or clinical population of interest?	Yes		
4. Were all eligible participants that met the prespecified entry criteria enrolled?			CD
5. Was the sample size sufficiently large to provide confidence in the findings?	Yes		
6. Was the test/service/intervention clearly described and delivered consistently across the study population?		No	
7. Were the outcome measures prespecified, clearly defined, valid, reliable, and assessed consistently across all study participants?	Yes		
8. Were the people assessing the outcomes blinded to the participants' exposures/interventions?		No	
9. Was the loss to follow-up after baseline 20% or less? Were those lost to follow-up accounted for in the analysis?		No	
10. Did the statistical methods examine changes in outcome measures from before to after the intervention? Were statistical tests done that provided p values for the pre-to-post changes?	Yes		
11. Were outcome measures of interest taken multiple times before the intervention and multiple times after the intervention (i.e., did they use an interrupted time-series design)?			NA
12. If the intervention was conducted at a group level (e.g., a whole hospital, a community, etc.) did the statistical analysis take into account the use of individual-level data to determine effects at the group level?			NA
**Quality Rating (Good, Fair, or Poor) (see guidance)**			
Rater #1 HS: Fair			
Rater #2 EL: Fair			
Additional Comments (If POOR, please state why):			

***CD, cannot determine; NR, not reported; NA, not applicable.**

Jeppesen (2015) [Systematic Review]

**Criteria**	**Yes**	**No**	**Other (CD, NR, NA)***
1. Is the review based on a focused question that is adequately formulated and described?	Yes		
2. Were eligibility criteria for included and excluded studies predefined and specified?	Yes		
3. Did the literature search strategy use a comprehensive, systematic approach?	Yes, searches were not that comprehensive, so, some limitations in the search strategy.		
4. Were titles, abstracts, and full-text articles dually and independently reviewed for inclusion and exclusion to minimize bias?			CD
5. Was the quality of each included study rated independently by two or more reviewers using a standard method to appraise its internal validity?		No	
6. Were the included studies listed along with important characteristics and results of each study?	Yes		
7. Was publication bias assessed?		No	
8. Was heterogeneity assessed? (This question applies only to meta-analyses.)			NA
**Quality Rating (Good, Fair, or Poor) (see guidance)**			
Rater HS: Fair			
Rater EL: Fair			
Additional Comments (If POOR, please state why):			

***CD, cannot determine; NR, not reported; NA, not applicable.**

Kilgour (2015)

Criteria	Yes	No	CD*
1. Was there a clear statement of the aims of the research?<br>• What was the goal of the research?<br>• Why is it thought important?<br>• Its relevance	Yes		
2. Is a qualitative methodology appropriate?<br>• If the research seeks to interpret or illuminate the actions and/or subjective experiences of research participants.<br>• Is qualitative research the right methodology for addressing the research goal?	Yes		
3. Was the research design appropriate to address the aims of the research?<br>• If the research has justified the research design (e.g. have they discussed how they decided which method to use)?	Yes		
4. Was the recruitment strategy appropriate to the aims of the research?<br>• If the researcher has explained how the participants were selected.<br>• If they explained why the participants they selected were the most appropriate to provide access to the type of knowledge sought by the study.<br>• If there are any discussions around recruitment (e.g. why some people chose not to take part)		No	
5. Was the data collected in a way that addressed the research issue?<br>• If the setting for data was justified<br>• If it is clear how data were collected (e.g. focus group, semi-structured interview etc.)<br>• If the researcher has justified the methods chosen.<br>• If the researcher has made the methods explicit (e.g. for interview method, is there an indication of how interviews were conducted, or did they use a topic guide)?<br>• If methods were modified during the study. If so has the researcher explained how and why?<br>• If the form of data is clear (e.g. tape recordings, video material, notes, etc.)<br>• If the researcher has discussed saturation of data.	Yes		
6. Has the relationship between researcher and participants been adequately considered?<br>• If the researcher critically examined their own role, potential bias and influence during (a) Formulation of the research questions (b) Data Collection, including sample recruitment and choice of location.<br>• How the researcher responded to events during the study and whether they considered the implications of any changes in the research design.		No	
7. Have ethical issues been taken in to consideration?<br>• If there are sufficient details of how the research was explained to participants for the reader to assess whether ethical standards were maintained.<br>• If the researcher has discussed issues raised by the study (e.g. issues around informed consent or confidentiality or how they have handled the effects of the study on the participants during and after the study)<br>• If approval has been sought form the ethics committee.	Yes		

8. Was the data analysis sufficiently rigorous?<br>• If there is an in-depth description of the analysis process<br>• If thematic analysis is used, if so is it clear how the categories/themes were derived from the data?<br>• Whether the researcher explains how the data presented were selected from the original sample to demonstrate the analysis process<br>• If sufficient data are presented to support findings<br>• To what extent contradictory data are taken into account<br>• Whether the researcher critically examined their own role, potential bias and influence during analysis and selection of data for presentation.	Yes		
9. Is there a clear statement of findings?<br>• If the findings are explicit<br>• If there is adequate discussion of the evidence both for and against the researchers' arguments<br>• If the researcher has discussed the credibility of their findings (e.g. triangulation, respondent validation, more than one analyst)<br>• If their findings are discussed in relation to the original research questions	Yes		
10. How valuable is the research?<br>• If the researcher discusses the contribution the study makes to existing knowledge or understanding e.g. do they consider the findings in relation to current practice or policy, or relevant research-based literature?<br>• If they identify new areas where research is necessary<br>• If the researchers have discussed whether or how the findings can be transferred to other populations or considered other ways the research may be used.	Yes		
**Quality Rating (Good, Fair, or Poor)**			
Rater #1 HS: Good			
Rater #2 EL: possibly good depending on final decision based on the comments above, if not will be happy with Fair			
Additional Comments (If POOR, please state why):			

***CD, cannot determine.**

Lie (2013)

Criteria	Yes	No	CD*
1. Was there a clear statement of the aims of the research?<br>• What was the goal of the research?<br>• Why is it thought important?<br>• Its relevance	Yes		
2. Is a qualitative methodology appropriate?<br>• If the research seeks to interpret or illuminate the actions and/or subjective experiences of research participants.<br>• Is qualitative research the right methodology for addressing the research goal?	Yes		
3. Was the research design appropriate to address the aims of the research?<br>• If the research has justified the research design (e.g. have they discussed how they decided which method to use)?		No	
4. Was the recruitment strategy appropriate to the aims of the research?<br>• If the researcher has explained how the participants were selected.<br>• If they explained why the participants they selected were the most appropriate to provide access to the type of knowledge sought by the study.<br>• If there are any discussions around recruitment (e.g. why some people chose not to take part)	Partial		
5. Was the data collected in a way that addressed the research issue?<br>• If the setting for data was justified<br>• If it is clear how data were collected (e.g. focus group, semi-structured interview etc.)<br>• If the researcher has justified the methods chosen.<br>• If the researcher has made the methods explicit (e.g. for interview method, is there an indication of how interviews were conducted, or did they use a topic guide)?<br>• If methods were modified during the study. If so has the researcher explained how and why?<br>• If the form of data is clear (e.g. tape recordings, video material, notes, etc.)<br>• If the researcher has discussed saturation of data.	Yes		
6. Has the relationship between researcher and participants been adequately considered?<br>• If the researcher critically examined their own role, potential bias and influence during (a) Formulation of the research questions (b) Data Collection, including sample recruitment and choice of location.<br>• How the researcher responded to events during the study and whether they considered the implications of any changes in the research design.		No	
7. Have ethical issues been taken in to consideration?<br>• If there are sufficient details of how the research was explained to participants for the reader to assess whether ethical standards were maintained.<br>• If the researcher has discussed issues raised by the study (e.g. issues around informed consent or confidentiality or how they have handled the effects of the study on the participants during and after the study)<br>• If approval has been sought form the ethics committee.	Partial		

8. Was the data analysis sufficiently rigorous?<br>• If there is an in-depth description of the analysis process<br>• If thematic analysis is used, if so is it clear how the categories/themes were derived from the data?<br>• Whether the researcher explains how the data presented were selected from the original sample to demonstrate the analysis process<br>• If sufficient data are presented to support findings<br>• To what extent contradictory data are taken into account<br>• Whether the researcher critically examined their own role, potential bias and influence during analysis and selection of data for presentation.	Yes (except last 2 bullet points)		
9. Is there a clear statement of findings?<br>• If the findings are explicit<br>• If there is adequate discussion of the evidence both for and against the researchers' arguments<br>• If the researcher has discussed the credibility of their findings (e.g. triangulation, respondent validation, more than one analyst)<br>• If their findings are discussed in relation to the original research questions	Yes		
10. How valuable is the research?<br>• If the researcher discusses the contribution the study makes to existing knowledge or understanding e.g. do they consider the findings in relation to current practice or policy, or relevant research-based literature?<br>• If they identify new areas where research is necessary<br>• If the researchers have discussed whether or how the findings can be transferred to other populations or considered other ways the research may be used.	Yes		
**Quality Rating (Good, Fair, or Poor)**			
Rater #1 HS: Fair			
Rater #2 JC: Fair			
Additional Comments (If POOR, please state why):			

***CD, cannot determine.**

Minsart (2014)

Criteria	Yes	No	CD*
1. Was there a clear statement of the aims of the research?<br>• What was the goal of the research?<br>• Why is it thought important?<br>• Its relevance	Yes		
2. Is a qualitative methodology appropriate?<br>• If the research seeks to interpret or illuminate the actions and/or subjective experiences of research participants.<br>• Is qualitative research the right methodology for addressing the research goal?	Yes		
3. Was the research design appropriate to address the aims of the research?<br>• If the research has justified the research design (e.g. have they discussed how they decided which method to use)?		No	
4. Was the recruitment strategy appropriate to the aims of the research?<br>• If the researcher has explained how the participants were selected.<br>• If they explained why the participants they selected were the most appropriate to provide access to the type of knowledge sought by the study.<br>• If there are any discussions around recruitment (e.g. why some people chose not to take part)	Yes		
5. Was the data collected in a way that addressed the research issue?<br>• If the setting for data was justified<br>• If it is clear how data were collected (e.g. focus group, semi-structured interview etc.)<br>• If the researcher has justified the methods chosen.<br>• If the researcher has made the methods explicit (e.g. for interview method, is there an indication of how interviews were conducted, or did they use a topic guide)?<br>• If methods were modified during the study. If so has the researcher explained how and why?<br>• If the form of data is clear (e.g. tape recordings, video material, notes, etc.)<br>• If the researcher has discussed saturation of data.	Yes		
6. Has the relationship between researcher and participants been adequately considered?<br>• If the researcher critically examined their own role, potential bias and influence during (a) Formulation of the research questions (b) Data Collection, including sample recruitment and choice of location.<br>• How the researcher responded to events during the study and whether they considered the implications of any changes in the research design.		No	
7. Have ethical issues been taken in to consideration?<br>• If there are sufficient details of how the research was explained to participants for the reader to assess whether ethical standards were maintained.<br>• If the researcher has discussed issues raised by the study (e.g. issues around informed consent or confidentiality or how they have handled the effects of the study on the participants during and after the study)<br>• If approval has been sought form the ethics committee.	Yes		

8. Was the data analysis sufficiently rigorous?<br>• If there is an in-depth description of the analysis process<br>• If thematic analysis is used, if so is it clear how the categories/themes were derived from the data?<br>• Whether the researcher explains how the data presented were selected from the original sample to demonstrate the analysis process<br>• If sufficient data are presented to support findings<br>• To what extent contradictory data are taken into account<br>• Whether the researcher critically examined their own role, potential bias and influence during analysis and selection of data for presentation.	Partial		
9. Is there a clear statement of findings?<br>• If the findings are explicit<br>• If there is adequate discussion of the evidence both for and against the researchers' arguments<br>• If the researcher has discussed the credibility of their findings (e.g. triangulation, respondent validation, more than one analyst)<br>• If their findings are discussed in relation to the original research questions	Yes		
10. How valuable is the research?<br>• If the researcher discusses the contribution the study makes to existing knowledge or understanding e.g. do they consider the findings in relation to current practice or policy, or relevant research-based literature?<br>• If they identify new areas where research is necessary<br>• If the researchers have discussed whether or how the findings can be transferred to other populations or considered other ways the research may be used.	Partial		
**Quality Rating (Good, Fair, or Poor)**			
Rater #1 HS: Good			
Rater #2 JC: Fair			
Additional Comments (If POOR, please state why):			

***CD, cannot determine.**

Nielsen (2014) [Systematic Review]

**Criteria**	**Yes**	**No**	**Other (CD, NR, NA)***
1. Is the review based on a focused question that is adequately formulated and described?		No	
2. Were eligibility criteria for included and excluded studies predefined and specified?			Partial
3. Did the literature search strategy use a comprehensive, systematic approach?	Yes		
4. Were titles, abstracts, and full-text articles dually and independently reviewed for inclusion and exclusion to minimize bias?			CD
5. Was the quality of each included study rated independently by two or more reviewers using a standard method to appraise its internal validity?		No	
6. Were the included studies listed along with important characteristics and results of each study?		No	
7. Was publication bias assessed?		No	
8. Was heterogeneity assessed? (This question applies only to meta-analyses.)			NA
**Quality Rating (Good, Fair, or Poor) (see guidance)**			
Rater #1 HS: Fair			
Rater #2 JC: Poor			
Additional Comments (If POOR, please state why):			

***CD, cannot determine; NR, not reported; NA, not applicable.**

Nielsen (2015)

Criteria	Yes	No	CD*
1. Was there a clear statement of the aims of the research?<br>• What was the goal of the research?<br>• Why is it thought important?<br>• Its relevance	Yes		
2. Is a qualitative methodology appropriate?<br>• If the research seeks to interpret or illuminate the actions and/or subjective experiences of research participants.<br>• Is qualitative research the right methodology for addressing the research goal?	Yes		
3. Was the research design appropriate to address the aims of the research?<br>• If the research has justified the research design (e.g. have they discussed how they decided which method to use)?		No	
4. Was the recruitment strategy appropriate to the aims of the research?<br>• If the researcher has explained how the participants were selected.<br>• If they explained why the participants they selected were the most appropriate to provide access to the type of knowledge sought by the study.<br>• If there are any discussions around recruitment (e.g. why some people chose not to take part)	Partial		
5. Was the data collected in a way that addressed the research issue?<br>• If the setting for data was justified<br>• If it is clear how data were collected (e.g. focus group, semi-structured interview etc.)<br>• If the researcher has justified the methods chosen.<br>• If the researcher has made the methods explicit (e.g. for interview method, is there an indication of how interviews were conducted, or did they use a topic guide)?<br>• If methods were modified during the study. If so has the researcher explained how and why?<br>• If the form of data is clear (e.g. tape recordings, video material, notes, etc.)<br>• If the researcher has discussed saturation of data.	Yes		
6. Has the relationship between researcher and participants been adequately considered?<br>• If the researcher critically examined their own role, potential bias and influence during (a) Formulation of the research questions (b) Data Collection, including sample recruitment and choice of location.<br>• How the researcher responded to events during the study and whether they considered the implications of any changes in the research design.	Yes		
7. Have ethical issues been taken in to consideration?<br>• If there are sufficient details of how the research was explained to participants for the reader to assess whether ethical standards were maintained.<br>• If the researcher has discussed issues raised by the study (e.g. issues around informed consent or confidentiality or how they have handled the effects of the study on the participants during and after the study)<br>• If approval has been sought form the ethics committee.	Yes		

8. Was the data analysis sufficiently rigorous?<br>• If there is an in-depth description of the analysis process<br>• If thematic analysis is used, if so is it clear how the categories/themes were derived from the data?<br>• Whether the researcher explains how the data presented were selected from the original sample to demonstrate the analysis process<br>• If sufficient data are presented to support findings<br>• To what extent contradictory data are taken into account<br>• Whether the researcher critically examined their own role, potential bias and influence during analysis and selection of data for presentation.	Yes, Contradictory, data not discussed.		
9. Is there a clear statement of findings?<br>• If the findings are explicit<br>• If there is adequate discussion of the evidence both for and against the researchers' arguments<br>• If the researcher has discussed the credibility of their findings (e.g. triangulation, respondent validation, more than one analyst)<br>• If their findings are discussed in relation to the original research questions	Yes		
10. How valuable is the research?<br>• If the researcher discusses the contribution the study makes to existing knowledge or understanding e.g. do they consider the findings in relation to current practice or policy, or relevant research-based literature?<br>• If they identify new areas where research is necessary<br>• If the researchers have discussed whether or how the findings can be transferred to other populations or considered other ways the research may be used.	Yes		
**Quality Rating (Good, Fair, or Poor)**			
Rater #1 HS: Good			
Rater #2 JC: Good			
Additional Comments (If POOR, please state why):			

***CD, cannot determine.**

Peticca (2014)

Criteria	Yes	No	Other (CD, NR, NA)*
1. Was the research question or objective in this paper clearly stated?	Yes		
2. Was the study population clearly specified and defined?	Yes		
3. Was the participation rate of eligible persons at least 50%?	Yes		
4. Were all the subjects selected or recruited from the same or similar populations (including the same time period)? Were inclusion and exclusion criteria for being in the study pre-specified and applied uniformly to all participants?	Yes		
5. Was a sample size justification, power description, or variance and effect estimates provided?		No	
6. For the analyses in this paper, were the exposure(s) of interest measured prior to the outcome(s) being measured?		No	
7. Was the timeframe sufficient so that one could reasonably expect to see an association between exposure and outcome if it existed?	Yes		
8. For exposures that can vary in amount or level, did the study examine different levels of the exposure as related to the outcome (e.g., categories of exposure, or exposure measured as continuous variable)?			NA
9. Were the exposure measures (independent variables) clearly defined, valid, reliable, and implemented consistently across all study participants?		No	
10. Was the exposure(s) assessed more than once over time?			NA
11. Were the outcome measures (dependent variables) clearly defined, valid, reliable, and implemented consistently across all study participants?	Yes		
12. Were the outcome assessors blinded to the exposure status of participants?		No	
13. Was loss to follow-up after baseline 20% or less?		No	
14. Were key potential confounding variables measured and adjusted statistically for their impact on the relationship between exposure(s) and outcome(s)?		No	
**Quality Rating (Good, Fair, or Poor) (see guidance)**			
Rater #1 HS: Fair			
Rater #2 EL: Poor			
Additional Comments (If POOR, please state why):			

***CD, cannot determine; NR, not reported; NA, not applicable.**

Sterne (2011)

Criteria	Yes	No	CD*
1. Was there a clear statement of the aims of the research?<br>• What was the goal of the research?<br>• Why is it thought important?<br>• Its relevance	Yes		
2. Is a qualitative methodology appropriate?<br>• If the research seeks to interpret or illuminate the actions and/or subjective experiences of research participants.<br>• Is qualitative research the right methodology for addressing the research goal?	Yes		
3. Was the research design appropriate to address the aims of the research?<br>• If the research has justified the research design (e.g. have they discussed how they decided which method to use)?		No	
4. Was the recruitment strategy appropriate to the aims of the research?<br>• If the researcher has explained how the participants were selected.<br>• If they explained why the participants they selected were the most appropriate to provide access to the type of knowledge sought by the study.<br>• If there are any discussions around recruitment (e.g. why some people chose not to take part)	Yes		
5. Was the data collected in a way that addressed the research issue?<br>• If the setting for data was justified<br>• If it is clear how data were collected (e.g. focus group, semi-structured interview etc.)<br>• If the researcher has justified the methods chosen.<br>• If the researcher has made the methods explicit (e.g. for interview method, is there an indication of how interviews were conducted, or did they use a topic guide)?<br>• If methods were modified during the study. If so has the researcher explained how and why?<br>• If the form of data is clear (e.g. tape recordings, video material, notes, etc.)<br>• If the researcher has discussed saturation of data.	Yes		
6. Has the relationship between researcher and participants been adequately considered?<br>• If the researcher critically examined their own role, potential bias and influence during (a) Formulation of the research questions (b) Data Collection, including sample recruitment and choice of location.<br>• How the researcher responded to events during the study and whether they considered the implications of any changes in the research design.		No	
7. Have ethical issues been taken in to consideration?<br>• If there are sufficient details of how the research was explained to participants for the reader to assess whether ethical standards were maintained.<br>• If the researcher has discussed issues raised by the study (e.g. issues around informed consent or confidentiality or how they have handled the effects of the study on the participants during and after the study)<br>• If approval has been sought form the ethics committee.	Yes		

8. Was the data analysis sufficiently rigorous?<br>• If there is an in-depth description of the analysis process<br>• If thematic analysis is used, if so is it clear how the categories/themes were derived from the data?<br>• Whether the researcher explains how the data presented were selected from the original sample to demonstrate the analysis process<br>• If sufficient data are presented to support findings<br>• To what extent contradictory data are taken into account<br>• Whether the researcher critically examined their own role, potential bias and influence during analysis and selection of data for presentation.		No	
9. Is there a clear statement of findings?<br>• If the findings are explicit<br>• If there is adequate discussion of the evidence both for and against the researchers' arguments<br>• If the researcher has discussed the credibility of their findings (e.g. triangulation, respondent validation, more than one analyst)<br>• If their findings are discussed in relation to the original research questions	Yes		
10. How valuable is the research?<br>• If the researcher discusses the contribution the study makes to existing knowledge or understanding e.g. do they consider the findings in relation to current practice or policy, or relevant research-based literature?<br>• If they identify new areas where research is necessary<br>• If the researchers have discussed whether or how the findings can be transferred to other populations or considered other ways the research may be used.	Yes		
**Quality Rating (Good, Fair, or Poor)**			
Rater #1 HS: Good			
Rater #2 JC: Fair			
Additional Comments (If POOR, please state why):			

***CD, cannot determine.**

Van Ryswyk (2015) [Systematic Review]

**Criteria**	**Yes**	**No**	**Other (CD, NR, NA)***
1. Is the review based on a focused question that is adequately formulated and described?	Yes		
2. Were eligibility criteria for included and excluded studies predefined and specified?	Yes		
3. Did the literature search strategy use a comprehensive, systematic approach?	Yes		
4. Were titles, abstracts, and full-text articles dually and independently reviewed for inclusion and exclusion to minimize bias?	Yes		
5. Was the quality of each included study rated independently by two or more reviewers using a standard method to appraise its internal validity?	Yes		
6. Were the included studies listed along with important characteristics and results of each study?	Yes		
7. Was publication bias assessed?		No	
8. Was heterogeneity assessed? (This question applies only to meta-analyses.)			NA
**Quality Rating (Good, Fair, or Poor) (see guidance)**			
Rater #1 HS: Good			
Rater #2 EL: Good			
Additional Comments (If POOR, please state why):			

***CD, cannot determine; NR, not reported; NA, not applicable.**

Van Ryswyk (2015)

Criteria	Yes	No	Other (CD, NR, NA)*
1. Was the study described as randomized, a randomized trial, a randomized clinical trial, or an RCT?	Yes		
2. Was the method of randomization adequate (i.e., use of randomly generated assignment)?	Yes		
3. Was the treatment allocation concealed (so that assignments could not be predicted)?	Yes		
4. Were study participants and providers blinded to treatment group assignment?		No	
5. Were the people assessing the outcomes blinded to the participants' group assignments?		No	
6. Were the groups similar at baseline on important characteristics that could affect outcomes (e.g., demographics, risk factors, co-morbid conditions)?	Yes		
7. Was the overall drop-out rate from the study at endpoint 20% or lower of the number allocated to treatment?	Yes		
8. Was the differential drop-out rate (between treatment groups) at endpoint 15 percentage points or lower?	Yes		
9. Was there high adherence to the intervention protocols for each treatment group?	Yes		
10. Were other interventions avoided or similar in the groups (e.g., similar background treatments)?	Yes		
11. Were outcomes assessed using valid and reliable measures, implemented consistently across all study participants?	Yes		
12. Did the authors report that the sample size was sufficiently large to be able to detect a difference in the main outcome between groups with at least 80% power?	Yes		

13. Were outcomes reported or subgroups analysed prespecified (i.e., identified before analyses were conducted)?	Yes		
14. Were all randomized participants analysed in the group to which they were originally assigned, i.e., did they use an intention-to-treat analysis?	Yes		
**Quality Rating (Good, Fair, or Poor) (see guidance)**			
Rater #1 HS: Good			
Rater #2 EL: Good			
Additional Comments (If POOR, please state why):			

***CD, cannot determine; NR, not reported; NA, not applicable.**

Van Ryswyk (2016)

**Criteria**	**Yes**	**No**	**Other (CD, NR, NA)***
1. Was the research question or objective in this paper clearly stated?	Yes		
2. Was the study population clearly specified and defined?	Yes		
3. Was the participation rate of eligible persons at least 50%?	Yes		
4a. Were all the subjects selected or recruited from the same or similar populations (including the same time period)?<br>4b. Were inclusion and exclusion criteria for being in the study prespecified and applied uniformly to all participants?	Yes<br><br>Yes		
5. Was a sample size justification, power description, or variance and effect estimates provided?		No	
6. For the analyses in this paper, were the exposure(s) of interest measured prior to the outcome(s) being measured?			NA
7. Was the timeframe sufficient so that one could reasonably expect to see an association between exposure and outcome if it existed?	Yes		
8. For exposures that can vary in amount or level, did the study examine different levels of the exposure as related to the outcome (e.g., categories of exposure, or exposure measured as continuous variable)?			NA
9. Were the exposure measures (independent variables) clearly defined, valid, reliable, and implemented consistently across all study participants?	Yes		
10. Was the exposure(s) assessed more than once over time?			NA
11. Were the outcome measures (dependent variables) clearly defined, valid, reliable, and implemented consistently across all study participants?			CD
12. Were the outcome assessors blinded to the exposure status of participants?		No	
13. Was loss to follow-up after baseline 20% or less?		No	
14. Were (a) key potential confounding variables measured and (b) adjusted statistically for their impact on the relationship between exposure(s) and outcome(s)?	Yes		
**Quality Rating (Good, Fair, or Poor) (see guidance)**			
Rater #1 HS: Good			
Rater #2 EL: Good			
Additional Comments (If POOR, please state why):			

***CD, cannot determine; NR, not reported; NA, not applicable.**
